# Paternity and kin structure among neighbouring groups in wild bonobos at Wamba

**DOI:** 10.1098/rsos.171006

**Published:** 2018-01-31

**Authors:** Shintaro Ishizuka, Yoshi Kawamoto, Tetsuya Sakamaki, Nahoko Tokuyama, Kazuya Toda, Hiroki Okamura, Takeshi Furuichi

**Affiliations:** 1 Primate Research Institute, Kyoto University, Inuyama, Aichi 484-8506, Japan; 2 Japan Society for the Promotion of Science, Kojimachi, Chiyoda-ku, Tokyo 102-0083, Japan; 3 Department of Evolutionary Studies of Biosystems, The Graduate University for Advanced Studies, Hayama, Kanagawa 240-0193, Japan

**Keywords:** bonobos, male philopatry, male–male reproductive competition, reproductive skew, genetic differentiation between groups, kin structure

## Abstract

Although both bonobos and chimpanzees are male-philopatric species, outcomes of male–male reproductive competition seem to be more closely associated with mating success in chimpanzees. This suggests that the extent of male reproductive skew is lower in bonobos. In addition, between-group male–male reproductive competition is more lethal in chimpanzees. This suggests that between-group differentiation in male kinship is lower in bonobos. We analysed the paternity of 17 offspring in two bonobo groups and estimated the relatedness of individuals among three neighbouring groups by using DNA extracted from non-invasive samples at Wamba, in the Democratic Republic of the Congo. The alpha males sired at least nine of 17 offspring. This supports a previous finding that the male reproductive skew is higher in bonobos than that in chimpanzees. Average relatedness among males within groups was significantly higher than that among males across groups, whereas there was no significant difference among females between within and across groups. These results are consistent with male philopatry, highly skewed reproductive success of males and female dispersal. Higher average relatedness among males within groups suggest that the differences in hostility towards males of different groups between bonobos and chimpanzees may be explained by factors other than kinship.

## 1. Introduction

Males of group-living animals compete to gain higher dominance ranks. This could be explained by the fact that higher dominance ranks are associated with greater reproductive success [1]. In cases where female receptivity is not asynchronous, the number of receptive females is limited at the same time, which enables a few males to monopolize breeding. Therefore, in various taxa of group-living animals, reproductive success is highly skewed towards males with higher dominance ranks [2–4]. Although our understandings of these systems mostly come from the previous studies on female-philopatric species, in which females remain in their natal groups and males disperse from their natal groups after reaching maturity, some species of group-living animals occur in male-philopatric societies (reviewed in [5]). In male-philopatric species, it is predicted that some males within groups are related by kinship. Therefore, males of male-philopatric species may be able to gain the inclusive fitness via the reproductive success of other males related to them [6]. This suggests that the mechanism of male–male reproductive competition may differ between male-philopatric species and female-philopatric species. For a more complete understanding of male–male reproductive competition in group-living animals, male-philopatric species should be investigated to clarify the mechanism of reproductive competition among males which are potentially related by kinship.

Comparisons between bonobos (*Pan paniscus*) and chimpanzees (*Pan troglodytes*) are effective for understanding male–male reproductive competition in male-philopatric species. These two species are the most closely related species with one another, and share many traits in terms of social systems, including male philopatry, female dispersal, multi-male/multi-female group composition and fission–fusion dynamics [7–10]. However, the intensity of male–male competition is remarkably different between these two species: aggression between chimpanzee males can be lethal and sometimes results in death, whereas that between bonobo males is more moderate and occurs less frequently [7,8,11,12]. One potential factor to explain the less intensive reproductive competition among bonobo males is the extended periods of female sexual receptivity and attractiveness [13]. This would modulate the intensity of male–male competition for mating opportunities and would increase the potential for female choice [14]. It follows, then, that the extent of reproductive skew among bonobo males is predicted to be lower than that among chimpanzee males. A recent study from LuiKotale reported that the male reproductive skew is higher in bonobos than in chimpanzees [15]. However, there have been many studies on the reproductive skew in chimpanzees compared with only two studies on the reproductive skew in bonobos [15,16]. It could be expected that comparing male reproductive skew between these two species could help to elucidate whether the intensity of male–male reproductive competition can lead to greater reproductive outcomes in male-philopatric species. Therefore, it is necessary to obtain more data on the reproductive skew among bonobo males.

To understand male–male reproductive competition, it is necessary to investigate kinship patterns among males. Kinship regulates affinity or antagonism in social interactions [17,18]. Therefore, kinship patterns among males could affect the intensity of reproductive competition. Although some males within groups could be related by kinship in male-philopatric species, some previous studies reported that the patterns of alliance formation or cooperative interactions among males were not explained by kinship (chimpanzees [19] and bottlenose dolphins [20]). However, between-group male-male reproductive competition potentially can be explained by kinship. Although both bonobos and chimpanzees are male-philopatric species and it is predicted that males are more closely related within groups than between groups, the antagonism towards males of different groups is completely different between these two species. For example, chimpanzee males sometimes make lethal coalitionary attacks and kill males of different groups, whereas bonobo males do not make such attacks [21–24]. This difference may be explained by the fact that between-group differentiation in male kinship is greater in chimpanzees than in bonobos. This prediction is supported by a previous study which showed that the estimated male-mediated gene flow was more frequent in bonobos than in chimpanzees [25]. However, although there have been a few studies on genetic differentiation between chimpanzee groups, there have been limited studies on genetic differentiation between bonobo groups [25–28]. Comparisons of between-group differentiation in male kinship may explain the differences in hostility towards males of different groups between these two species. Thus, further investigation on kinship both within and across bonobo groups is necessary to elucidate this.

This study aimed to reveal kin structure among three neighbouring groups in wild bonobos at Wamba in the Luo scientific reserve of the Democratic Republic of the Congo, where intensive research based on individual identification has continued since 1976. At first, we evaluated the reproductive skew among bonobo males in two neighbouring groups. Second, we estimated the pairwise relatedness among all pairs of individuals among three neighbouring groups and compared the average relatedness within groups with that across groups.

## 2. Material and Methods

### 2.1. Study subjects and DNA sampling

Study subjects were three neighbouring bonobo groups (E1, PE and PW) at Wamba in the Luo Scientific Reserve area of the Democratic Republic of the Congo [28]. Table 1 summarizes the composition of each group. We categorized age classes of individuals defined in a previous study [29]: adult males (age > 15 years), adolescent males (age > 8 years), immigrant parous females, immigrant nulliparous females, and infants and juveniles (both sexes age ≦ 8 years). The E group has been studied since 1974, which split into the E1 and E2 groups by 1983 [30]. Artificial food provisioning had been conducted intermittently to intensively observe behaviours of animals until 1996. Between 1996 and 2003, a civil war prevented research in this area. After research resumed in 2003, animals have been observed in their natural conditions without artificial provisioning [31]. All individuals of the E1 group have been well habituated and identified since 1976. In 2014, this group consisted of 37 individuals including 10 adult and adolescent males and nine immigrant parous females. The P group was recognized in 1976 as a neighbouring group of the E group [24,32]. In 2010, one group, whose range was in the eastern part of the range used by the P group in the past, was named as the PE group. Another group, which has been neighbouring the PE group on its west side, was named as the PW group. Intensive research on the PW group was started at that time. All individuals of the PE group have been identified since 2011. In 2014, this group consisted of 26 individuals including six adult and adolescent males and nine females which have been estimated as immigrant and parous. The E1 and PE groups have been continuously followed from bed to bed for at least 5 years. Field research for the PW group has been carried out sporadically since 2011 (almost once per month). All individuals of this group have been identified since 2012. In 2014, this group consisted of 14 individuals including five adult and adolescent males and four females, which have been estimated as immigrant and parous during the sampling period. For DNA analysis, faeces and urine were collected from 2007 to 2016. Most samples were collected by S.I. in July 2015, and from December 2015 to February 2016. Faecal samples were collected using cotton swabs, and urine samples were collected using a disposable plastic syringe. Both types of collected samples were conserved in lysis buffer at normal temperature [33,34]. A total of 86 faecal and seven urine samples were used for analysis.

**Table 1.** Composition of each group when the kin structure was investigated.

	E1	PE	PW
adult males	7	5	1
adolescent males	3	1	4
immigrant parous females	9	9	4
immigrant nulliparous females	4	0	2
infants and juveniles	14	11	3
total	37	26	14

### 2.2. Dominance relationships among males

The dominance ranks of males of the E1 and PE groups were classified based on the value of normalized David's score [35]. Dyadic aggressive interactions among adult and adolescent males of the E1 group were recorded by trained local assistants, and that value for each male was calculated each year from 2010 to 2013. Dyadic aggressive interactions among adult and adolescent males of the PE group were recorded by the authors and local assistants, and that value for each male was calculated. The males whose values were the highest were categorized as the alpha. The linearity of each dominance hierarchy was calculated using Laundau's modified *h*′. The value was calculated and its significance was checked, using the R package ‘Compete v. 0.1’.

### 2.3. Genotyping

Following a previous study, DNA was extracted from the non-invasively collected samples [33]. After removing extra components in the lysate by centrifugation at 10 000*g* for 10 min, 600 µg of hydrolysed starch powder was added to the supernatant and then vortexed for 1 min to absorb inhibitors. The obtained supernatant was transferred to a plastic tube and cleaned using a commercially available silica column system (Wizard SV Gel and PCR Clean-Up System, Promega, Madison, IW, USA). For genotyping, we analysed eight autosomal short tandem repeats [36–38]. We prepared two primer sets: set A (D6S493, D7S817 and D9S910) and set B (D2S1329, D3S1766, D3S1768, D4S1627 and D12S66) to carry out two-step multiplex PCR [39].

To check applicability of the obtained DNA, we followed the verification system designed in a previous study [33]. Semi-quantitatively, DNA from the obtained samples and human placenta (SIGMA) as a positive control with known concentration was amplified targeting the c-*myc* proto-oncogene [40]. By performing electrophoresis and comparing the intensity of PCR bands, we categorized the quantity of the samples: greater than 300 pg µl^−1^, greater than 100 pg µl^−1^ and less than or equal to 100 pg µl^−1^.

As the first step of two-step PCR genotyping, multiplex PCRs were performed in 20 μl reaction volumes comprising 1 µl of extracted faecal DNA or DNA mixture, 200 nM of each forward and reverse non-labelled primer (set A or B), 2× buffer, 0.4 mM of each dNTP and 0.5 U of KOD FX polymerase (Toyobo). Amplification parameters were as follows: 94°C for 5 min; 35 cycles of 94°C for 30 s, 64°C for 30 s and 68°C for 1 min; and a final 5 min extension at 66°C. Second, we carried out singleplex PCR amplifications. Singleplex PCRs were performed in 13 μl reaction volumes comprising 1 µl of multiplex PCR products, 50–100 nM of forward primer, fluorescently labelled by FAM, NED or HEX, and of reverse primer, 2× buffer, 0.4 mM of each dNTP and 0.5 U of KOD FX polymerase. Amplification parameters were as follows: 94°C for 5 min; 45 cycles of 94°C for 30 s, 64°C for 30 s and 68°C for 1 min; and a final 5 min extension at 66°C.

The amplification products were separated using capillary electrophoresis, using an ABI 3130xl Genetic Analyzer (Applied Biosystems, CA, USA). Alleles were sized using GeneMapper software v. 4.1 (Applied Biosystems).

Because DNA extracted from non-invasively collected samples is typically degraded and low in concentration, we repeated genotyping, following the recommendations of previous studies to ensure accurate genotyping [34,39–41]. At each locus, we identified homozygotes when the single allele was repeatedly observed at least three times in samples whose concentrations were more than 100 pg µl^−1^, and at least four times in samples whose concentrations were less than 100 pg µl^−1^. We identified heterozygotes when each allele was repeatedly observed more than twice at each locus.

### 2.4. Paternity analysis

The paternity of 17 out of 20 offspring born in the E1 and PE groups from 2009 to 2014 was investigated. Paternity was assigned by exclusion. First, by comparing the mother and offspring genotypes, the paternal alleles of the offspring were deduced. If candidate fathers did not possess the paternal alleles, paternity was excluded. Paternal candidates were all adult and adolescent males within each group and when all of them were excluded, males outside each group were added. When one candidate was not excluded, the confidence level for assignments was analysed by a likelihood method, using CERVUS [42]. Allele frequency was calculated for the three groups. To simulate the paternity analysis, we assumed that mothers were known, the proportion of loci mistyped was 0.01 and the error rate in likelihood was 0.01. The proportion of sampled candidates was estimated based on the number of existing candidate males when the offspring was conceived. When the most likely fathers had no mismatched alleles and the confidence level for the assignments was more than 99%, the male was concluded to be the father of the offspring. Based on the results of paternity of offspring in the E1 group, we calculated the Nonac's *B* index of reproductive skew [43].

### 2.5. Estimating pairwise relatedness

We estimated the pairwise relatedness values for all pairs of 43 individuals, including adult and adolescent males and immigrant parous females (10 males and nine females in the E1, six males and nine females in the PE, and five males and four females in the PW) by using the Queller and Goodnight estimator implemented in GeneAlEx6.3 [44]. The programme estimates relatedness values by allele sharing of pairs and allele frequency in the population. Allele frequency was calculated for all individuals used for estimating relatedness values in the three groups. To compare the average relatedness value for each category, a permutation test was conducted in R v. 3.31. A *p*-value was obtained by 999 permutations.

## 3. Results

### 3.1. Paternity analysis

Sixty-eight individuals in total were analysed at eight loci (electronic supplementary material, appendix). In the E1 group, the paternity of 10 offspring born between 2009 and 2013 was analysed and seven of them were assigned to single paternal candidates within each group, with a greater than 99% confidence level (table 2). Six out of seven offspring, whose paternity was assigned, were sired by the male NB. From 2010 to 2014, the dominance hierarchy of males was significantly linear and NB was the alpha male during that period (electronic supplementary material, table S1). Therefore, it can be considered that NB sired at least six offspring during his alpha period. In addition, Ki, who is the mother of KY and Kx, was also confirmed as the mother of NB by genetic analysis and demographic data [45]. Therefore, the reason why the father of KY was not NB might be NB's avoidance to mate with his mother Ki. The Nonac's *B* index of reproductive skew in the E1 group was 0.51. The paternity of three offspring was not successfully assigned to any paternal candidates analysed within this group. When these offspring were conceived, two other mature males belonged to this group, but disappeared later. Although they were paternal candidates of the three offspring, DNA was not collected from them and analysed. Therefore, these offspring were sired by such unsampled males or extra-group males.

**Table 2.** Paternity of offspring in the E1 group. (Nmis, number of mismatches with the most likely father.)

birth of time	estimated year of conception	offspring	mother	most likely father	alpha male at the time of conception	Nmis	Δ score	confidence level	no. of unsampled candidates
2009.6	2008	KY	Ki	—	?	3	—	—	2
2009.8	2009	HC	Hs	—	?	1	—	—	2
2009.10	2009	Ym	Yk	—	?	2	—	—	2
2010.12	2010	Fa	Fk	NB	NB	0	2.77	>99%	0
2010.12	2010	Ok	Ot	NB	NB	0	3.38	>99%	0
2011.12	2011	SE	Sl	NB	NB	0	3.59	>99%	0
2012.1	2011	Jl	Jk	NB	NB	0	2.74	>99%	0
2013.7	2012	NI	Nv	NB	NB	0	5.12	>99%	0
2014.1	2013	Kx	Ki	JD	NB	0	5.10	>99%	0
2014.2	2013	Ha	Hs	NB	NB	0	3.57	>99%	0

In the PE group, the paternity of seven offspring born from 2011 to 2014 was analysed and six of them were assigned to single paternal candidates within each group, with a greater than 99% confidence level (table 3). Four out of six assigned offspring were sired by SN. From 2012 to 2014, although the dominance hierarchy was not significantly linear, SN had been the alpha male (electronic supplementary material, table S2). Therefore, it can be considered that SN sired at least three offspring during his alpha period. The paternity of offspring, not assigned to SN, was assigned to different males. The paternity of one offspring was not successfully assigned to any paternal candidates analysed within this group. When this offspring was conceived, one more mature male belonged to this group but disappeared later. Although he was a paternal candidate of such offspring, DNA from him was not collected and analysed. Therefore, this offspring was sired by an unsampled male or extra-group males. In the time period for which information of dominance relationships between males and all the present males at conception is available, nine out of 11 offspring were sired by the alpha males and the most successful sire's share was in total 81.8%. Although there were no absolute cases of extra-group paternity, four offspring, whose paternity was not assigned, might have been sired by unsampled males within each group or extra-group males.

**Table 3.** Paternity of offspring in the PE group. (Nmis, number of mismatches with the most likely father.)

birth of time	estimated time of conception	offspring	mother	most likely father	alpha male at the time of conception	Nmis	Δ score	confidence level	no. of unsampled candidates
2011.2	2010	HO	Hd	SN	?	0	5.45	>99%	1
2012.3	2011	KL	Kb	—	?	4	—	—	1
2012.3	2011	IA	Ic	ML	?	0	9.79	>99%	0
2012.11	2012	Mz	Mt	SN	SN	0	6.94	>99%	0
2013.3	2012	Pk	Po	SN	SN	0	7.56	>99%	0
2013.5	2012	So	Sk	SN	SN	0	10.95	>99%	0
2014.7	2013	Ma	Mr	GI	SN	0	3.52	>99%	0

### 3.2. Pairwise relatedness value of adult and adolescent males and immigrant parous females

**Figure 1.**
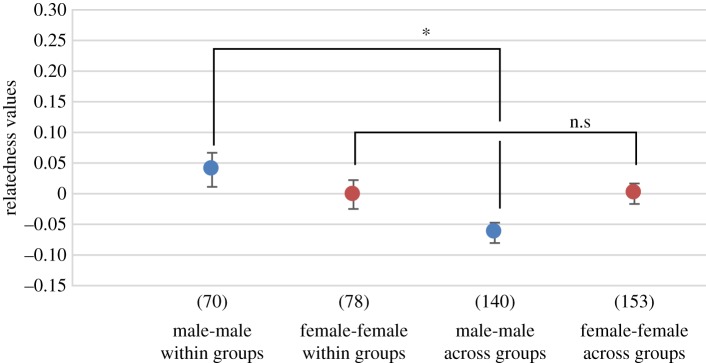
Average relatedness values (*R*) and standard errors for pairs of individuals in each category. Blue dots represent the average relatedness value among males within and across groups. Red dots represent the average relatedness among females within and across groups. The number of pairwise comparisons used to calculate each value appears in brackets.

Because tests of deviation from Hardy and Weinberg equilibrium did not show a significant value at each locus, we pooled the three groups into one population (*χ*^2^-test, *p* < 0.01). Average relatedness among males within groups was significantly higher than that across groups (permutation test, *p* < 0.01; figure 1). The average relatedness value between males of the PE group and those of the PW group was relatively high and significantly higher than those between males of the E1 group and those of the PE group (permutation test, *p* < 0.05; table 4). On the other hand, there was no significant difference among females between within and across groups (permutation test, *p*  =  0.936; figure 1).

**Table 4.** Average relatedness values within each group and pair of two groups.

among males/ among females	E1	PE	PW
E1	0.0213/−0.0461	—	—
PE	−0.0879/0.0055	0.0099/0.0203	—
PW	−0.0801/0.0074	0.0042/−0.0190	0.1905/0.1291

## 4. Discussion

**Figure 2.**
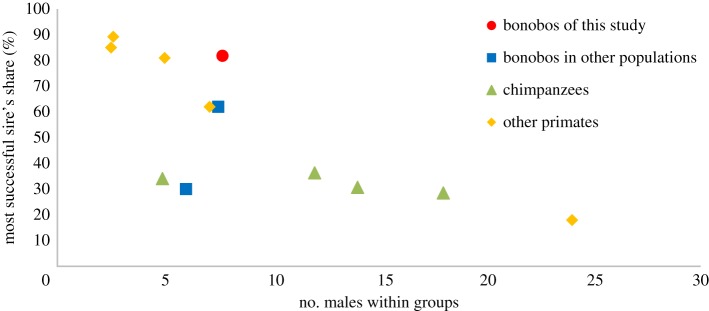
The most successful sire's share of multi-male/multi-female group-living primate species. Round, square, triangle and diamond dots represent the most successful sire's share in bonobos of this study, bonobos in other populations, chimpanzees and other primate species, respectively. Details on species, population and publications are described in the electronic supplementary material, table S3.

Nine out of 11 offspring in the two study groups were sired by the alpha males and the percentage of the most successful sire's share was in total 81.8%, in the time period for which information on dominance relationships between males and all the present males at conception is available (E1: 86% and PE: 75%). The percentage of the most successful sire's share in the present study was higher than that in other multi-male/multi-female group-living primates (figure 2; electronic supplementary material, table S3; [15,16,46–54]). The percentage of the most successful sire's share in the two groups, as well as the Nonac's *B* index in the E1 group, was higher than that recorded in the Bompusa community at LuiKotale (most successful sire's share: 62% and Nonac's *B* index: 0.22 [15]). Although differences in these indices between the two study sites may be caused by some factors, such as the strength of maternal influences (discussed as below) or the stability of dominance relationships between males, at least both these results indicate that the male reproductive skew is higher in bonobos than in chimpanzees.

One reason why the alpha males were able to sire many offspring might be because they enjoy a higher mating success with strong support from their mothers. In bonobos, the alpha males tend to be sons of high-ranking females, as in the case of NB who was a son of Ki, an old and high-ranking female in at least the E1 group [7]. In bonobo society, old females tend to occupy a high social status and can defeat males in aggressive interactions [32]. Therefore, if males attack the alpha males, it could lead to the risk that they might be attacked and scattered by the alpha males' mothers. That is why males may give up attacking the alpha males. Instead, the alpha males may prevent other males from attacking them by always ranging with their mothers and thus enjoy high mating success. This is supported by a previous study, showing that mothers and sons have high association rates and mothers provide agonistic aid to sons in conflict with other males [55]. It could be assumed that such a mechanism produces a high paternity rate for the alpha males.

The paternity of four offspring was not assigned to analysed candidate fathers within the groups, suggesting that these offspring might be sired by either males of other groups, or those who looked like non-alpha males according to general observations and had disappeared later before the DNA sampling in this study (two males in the E1 group and one male in the PE group). If these offspring were sired by extra-group males, that would be consistent with frequently observed mating among different group members during group encounters [24]. A previous study reported that some of male-mediated gene flow might be produced by extra-group paternity [25]. This suggests that it is probable that some offspring in each group might be sired by extra-group males. On the other hand, if they were sired by unsampled males within the groups and there were few offspring sired by extra-group males, frequent mating between males and females from different groups did not seem to lead to conception as expected by its high frequency. In bonobos, females can mate with males even if they are not in a fertile period [13]. In addition, sexual interactions have other functions besides reproduction such as reducing tension [56]. Mating among different group individuals during group encounters may function for such purposes during group encounters and might not necessarily involve females in fertile periods. By continuing to investigate the paternity of offspring whose candidate fathers’ samples are completely sampled, the presence or absence of extra-group paternity is expected to become clear.

Average relatedness among males within groups was significantly higher than that across groups. This difference might be caused by male philopatry and skewed reproductive success of a limited number of males with alpha status. Although male gene flow between groups could occur because of some rare reasons such as extra-group paternity, immigration of females with dependent male offspring or even adolescent or adult male dispersal, these factors might not be sufficient to eliminate the differentiation in average relatedness of males between groups [25,57]. In addition, according to long-term observations at Wamba, males do not migrate between groups, and pregnant females or independent infants also do not migrate between groups [58]. These observations might explain the significant difference between average relatedness among males within groups and that across groups in our data.

Higher relatedness between males of the PE group and those of the PW group might reflect a closer relationship among these two groups in the past. Although it is confirmed that the E1 group has existed for at least 30 years as an independent group, members of the PE group were fully identified only in 2011 and those of the PW group in 2012. Therefore, there is a possibility that the PE and PW groups had split from one group more recently than 30 years ago. Another possibility is that such a structure might be caused by reproduction among members of these groups. The frequency of inter-group encounters, which may be connected to opportunities for reproduction, is much higher between these two adjacent groups than the E1 and PE groups, or the E1 and PW groups [59].

Higher average relatedness among males within groups was also observed in a previous study on chimpanzees [26]. In Tai forest, average relatedness among chimpanzee males within groups tends to be higher than that across groups. Although the available data are still limited, these suggest that between-group differentiation in male kinship is similar in bonobos and chimpanzees. Therefore, it might be impossible to explain differences in hostility towards males of different groups between bonobos and chimpanzees based purely on kinship. On the other hand, one study on western gorillas reported that males of neighbouring groups have kinship with each other, whereas another study reported that the single males leading groups were usually related to one or more nearby males [60,61]. Most western gorilla males emigrate while they are blackbacks or young silverbacks [62]. The between-group differentiation in male kinship in both bonobos and chimpanzees could be attributed to male philopatry, which is one of stable traits in the genus *Pan*.

There was no significant difference in average relatedness among females between within and across groups. The absence of significant difference in average relatedness among females between within and across groups was also reported in a previous study on chimpanzees [26]. This is consistent with the prediction of female dispersal in the present study. Although a recent study reported that there were two confirmed mother–daughter dyads within the group, the effects of this on kin structure might be limited to increasing the average relatedness among females within groups [63]. Our data also suggest that it is rare for females, which are related by kinship, to migrate and settle into the same group together. In other words, females who have settled into the same group may have come from various natal groups. This is supported by a high diversity of mitochondria DNA haplotypes within the groups [16,63,64]. In addition, our long-term observations showed that no females migrated into neighbouring groups until 2012 [58]. This suggests that it might be rare for females to migrate and settle into neighbouring groups.

This study found inconsistency between the highly skewed reproductive success of males and non-intense male–male reproductive competition in bonobos. To explain such an inconsistency, quantitative data should be collected to investigate the effects of female control, such as maternal influences, on the reproductive skew among males. This study also found evidence of between-group differentiation in male kinship in bonobos. However, because data on genetic differentiation between groups are still scarce for bonobos, it is expected that more data will be reported from other sites.

## Supplementary Material

Appendix; Tables S1 - S3

## Data accessibility

The datasets supporting this article have been uploaded as the part of the electronic supplementary material.
