# Supplementary material for: Paternity and kin structure among neighbouring groups in wild bonobos at Wamba
Source: R Soc Open Sci. 2018 Jan 31;5(1):171006. doi: 10.1098/rsos.171006 (PMC5792889; doi:10.1098/rsos.171006)
Supplement: Appendix; Tables S1 - S3 [file rsos171006supp1.pdf]

Appendix: Genotype data for individuals of the three groups

Name	Sex	Group	Age class	D6S493		D9S910		D7S817		D12S66		D3S1766		D4S1627		D2S1329		D3S1768	
Ki	F	E1	Immigrant parous	227	231	106	109	170	170	154	158	222	226	190	214	197	201	176	192
NB	M	E1	Adult	227	231	106	109	170	174	150	158	218	222	190	214	197	201	188	192
KT	M	E1	Adolescent	227	231	103	109	170	174	154	158	218	226	190	202	201	205	176	192
KY	M	E1	Juvenile	227	227	106	109	162	170	154	158	218	226	-	-	201	201	192	196
Kx	F	E1	Infant	227	235	103	109	170	174	150	154	222	226	190	214	201	201	176	192
No	F	E1	Immigrant parous	227	235	106	115	170	174	146	146	222	226	194	194	201	205	172	188
Hs	F	E1	Immigrant parous	223	235	106	106	174	182	146	150	222	222	190	190	201	201	184	188
HC	M	E1	Juvenile	231	235	106	106	174	174	150	150	218	222	190	194	197	201	188	188
Ha	F	E1	Infant	231	235	106	106	170	182	146	158	222	222	-	-	201	201	184	192
Jk	F	E1	Immigrant parous	239	239	103	103	166	174	146	154	218	218	202	214	201	205	188	188
JR	M	E1	Adolescent	231	239	103	103	166	174	130	146	218	218	194	202	201	205	188	200
JO	M	E1	Juvenile	231	239	103	103	166	166	154	154	214	218	190	214	197	201	188	188
Jl	F	E1	Infant	227	239	103	106	170	174	154	158	-	-	214	214	201	201	-	-
Sl	F	E1	Immigrant parous	231	235	106	118	170	178	142	150	222	222	194	194	197	205	180	188
SB	M	E1	Adolescent	231	231	103	106	178	178	150	158	215	222	194	214	197	201	188	192
SE	M	E1	Infant	227	235	109	118	170	178	142	150	218	222	190	194	197	201	188	188
Nv	F	E1	Immigrant parous	227	231	103	109	162	182	150	158	215	218	190	194	201	205	176	192
Nl	M	E1	Infant	227	227	106	109	162	170	158	158	218	218	194	214	201	201	188	192
Yk	F	E1	Immigrant parous	231	235	103	109	166	178	146	150	222	226	194	218	201	205	176	188
Ym	F	E1	Juvenile	231	243	106	109	166	174	150	158	222	226	190	194	201	205	188	196
Ot	F	E1	Immigrant parous	227	231	106	118	166	166	150	150	222	226	202	214	201	201	192	196
Ok	F	E1	Juvenile	227	231	106	118	166	174	150	158	218	226	190	214	201	201	188	196
Fk	F	E1	Immigrant parous	231	239	106	109	162	166	146	154	222	222	194	202	201	201	188	200
Fa	F	E1	Infant	227	231	109	109	166	174	146	158	-	-	-	-	197	201	188	200
Zn	F	E1	Immigrant nulliparous	235	243	112	112	166	166	150	154	218	222	210	210	201	201	188	188
Pf	F	E1	Immigrant nulliparous	227	227	112	118	166	170	146	150	222	226	190	194	197	197	184	188
Ik	F	E1	Immigrant nulliparous	227	243	106	106	170	174	146	150	222	226	190	194	197	209	188	192
Sc	F	E1	Immigrant nulliparous	231	235	106	118	170	174	146	146	222	226	194	210	197	205	176	188
TW	M	E1	Adult	227	235	106	109	166	170	146	150	222	226	194	206	201	201	184	192
TN	M	E1	Adult	227	231	106	106	166	174	158	158	218	222	202	206	197	201	176	188
GC	M	E1	Adult	227	231	103	106	174	178	158	158	215	218	202	214	201	205	176	192
DI	M	E1	Adult	231	235	103	106	166	174	146	154	214	226	190	194	197	201	184	188
LB	M	E1	Adult	235	235	103	109	166	166	146	154	218	227	190	194	201	205	176	192
JD	M	E1	Adult	227	235	103	106	166	174	140	150	222	226	190	194	197	201	176	192
Bk	F	PE	Immigrant parous	227	231	106	109	170	182	142	146	218	226	202	206	201	201	176	196
Kb	F	PE	Immigrant parous	227	235	103	106	162	166	146	154	222	226	190	206	197	201	188	188
KL	M	PE	Juvenile	235	243	106	106	162	178	146	154	226	226	190	194	197	197	176	188
Hd	F	PE	Immigrant parous	223	235	106	106	162	178	146	146	218	222	194	202	197	205	188	188
HO	M	PE	Juvenile	223	231	106	106	170	178	142	146	222	226	-	-	197	205	188	196
Sk	F	PE	Immigrant parous	231	231	106	118	170	174	142	146	222	222	190	210	197	201	188	196
So	F	PE	Infant	223	231	106	106	170	182	142	142	222	227	194	210	197	201	192	196
Po	F	PE	Immigrant parous	227	235	106	118	166	166	146	162	222	222	190	218	197	201	188	188
Pp	F	PE	Juvenile	227	231	106	106	166	182	142	162	222	226	190	214	197	205	188	196
Pk	F	PE	Infant	223	227	106	118	166	170	146	146	222	226	206	218	197	201	188	196
Mt	F	PE	Immigrant parous	227	243	103	109	170	178	146	150	226	226	194	218	197	201	180	196
Mz	F	PE	Infant	223	243	103	106	170	178	146	150	226	226	194	206	197	201	196	196

Appendix: To be continued

Name	Sex	Group	Age class	D6S493		D9S910		D7S817		D12S66		D3S1766		D4S1627		D2S1329		D3S1768	
Ic	F	PE	Immigrant parous	227	239	106	106	170	178	150	158	222	226	190	190	201	209	188	192
IR	M	PE	Adolescent	235	239	106	109	178	182	146	158	226	226	190	218	201	209	188	188
IA	M	PE	Infant	227	243	106	109	166	170	150	154	218	226	190	194	205	209	176	188
Mr	F	PE	Immigrant parous	227	237	106	109	162	174	150	154	222	227	190	190	201	205	176	196
Ma	F	PE	Infant	227	237	109	109	166	174	146	154	-	-	190	218	201	205	176	188
Nr	F	PE	Immigrant parous	227	239	109	118	162	174	146	150	222	222	190	190	205	205	188	188
SN	M	PE	Adult	223	231	106	106	170	182	142	146	226	227	194	206	201	205	192	196
TK	M	PE	Adult	227	235	103	106	162	170	146	146	218	222	194	206	201	205	188	192
ML	M	PE	Adult	239	243	109	109	166	166	146	154	218	222	194	194	205	205	176	192
DN	M	PE	Adult	227	235	103	106	162	170	146	154	222	226	190	194	201	205	176	188
GI	M	PE	Adult	235	237	109	109	166	174	146	154	226	227	190	218	201	205	176	188
Dk	F	PW	Immigrant parous	235	235	103	106	166	170	146	150	218	226	190	218	201	205	176	188
Db	F	PW	Infant	227	235	106	109	166	170	146	154	218	226	190	190	197	205	176	184
Ja	F	PW	Immigrant parous	235	237	106	106	170	174	146	154	218	222	190	194	201	205	176	196
Jj	F	PW	Infant	227	237	106	112	166	174	146	150	218	226	194	194	205	205	176	184
Fo	F	PW	Immigrant parous	227	235	103	103	162	174	146	150	214	218	190	194	197	201	192	192
Co	F	PW	Immigrant parous	235	235	103	109	170	170	146	158	222	222	190	194	201	201	176	176
WA	M	PW	Adult	227	235	109	112	166	170	150	154	218	226	190	194	197	205	184	192
TY	M	PW	Adolescent	227	231	109	118	162	170	154	154	226	226	194	194	197	205	184	188
BB	M	PW	Adolescent	227	235	106	109	170	174	154	154	218	218	194	194	205	205	192	196
DD	M	PW	Adolescent	239	243	106	109	162	166	146	158	214	218	194	194	197	205	172	188
DA	M	PW	Adolescent	227	235	103	109	166	170	146	150	226	226	190	190	197	205	172	184

Table S1: Dominance rank, number of dyadic aggressions, and h’ index of males in the E1 group in each year

	2010	2011	2012	2013
NB	1	1	1	1
TN	2	2	5	2
DI	3	3	2	6
LB	4	5	4	4
GC	5	4	3	3
JD	6	7	7	7
JR	7	8	6	5
TW	8	6	8	9
KT	-	-	-	8
SB	-	-	-	10
Total number of dyadic aggressions	144	123	256	110
h’	0.929**	0.834**	0.857**	0.612*

*: $p < 0.05$, **: $p < 0.01$

Table S2: Dominance rank, number of dyadic aggressions, and h’ index of males in the PE group in each year

	2012	2013
SN	1	1
TK	2	3
DN	3	5
ML	4	2
GI	5	4
IR	-	6
Total number of dyadic aggressions	74	127
h‘	1	0.772

*: $p < 0.05$, **: $p < 0.01$

Table S3: Average number of males within groups and most successful sire’s share in multi-male/multi-female group-living primates

Species	Average number of males within groups	Most successful sire’s share (%)	Reference
Bonobo (Wamba)	7.7	81.8	This study
Bonobo (LuiKotale)	7.4	62	[15]
Bonobo (Lomako)	6	30	[16]
Chimpanzee (Tai)	4.9	34.1	[46]
Chimpanzee (Gombe)	18	28.5	[47]
Chimpanzee (Mahale)	12	36.3	[48]
Chimpanzee (Budongo)	14	30.7	[49]
Mountain gorilla	2.5	85	[50]
Yellow baboon	5	81	[51]
White faced capuchin	7.3	62	[52]
Mousetached tamarin	2.6	89.2	[53]
Northern muriqui	24	18	[54]
